# Pan-Cancer Analysis Reveals the Potential of PLOD1 as a Prognostic and Immune Biomarker for Human Cancer

**DOI:** 10.3390/biomedicines12122653

**Published:** 2024-11-21

**Authors:** Zhao Zhai, Shuo Wang, Yudong Cao, Jia Liu, Qiang Zhao, Yongpeng Ji, Xiao Yang, Xingxing Tang, Jinchao Ma, Peng Du

**Affiliations:** Key Laboratory of Carcinogenesis and Translational Research (Ministry of Education/Beijing), Department of Urology, Peking University Cancer Hospital & Institute, Beijing 100089, China; zhaizhao0308@163.com (Z.Z.); wangshuoarea@pku.org.cn (S.W.); ydcao@bjmu.edu.cn (Y.C.); liujiabdyy@163.com (J.L.); pkuch_zhaoq@126.com (Q.Z.); jiyongpeng@163.com (Y.J.); 18611979333@163.com (X.Y.); xingxing.tang@bjcancer.org (X.T.); ma15565252290@163.com (J.M.)

**Keywords:** PLOD1, pan-cancer analysis, biomarker

## Abstract

**Background/Objectives:** Procollagen-lysine, 2-oxoglutarate 5-dioxygenase 1 (PLOD1) is known as an enhancer of collagen fiber deposition and cross-linking stability. However, there is limited information on its function in tumors. In this study, we aimed to elucidate the function and potential mechanism of action of PLOD1 across cancers. **Methods:** We assessed the pan-cancer expression, mutation, methylation and prognostic value of PLOD1 through multiple online databases. In addition, we performed correlation analyses of its immunological features, as well as functional assessment analyses of PLOD1. Finally, we assessed the effect of PLOD1 knockdown on bladder tumor cells using in vitro experiments. **Results:** Our findings suggest that PLOD1 is aberrantly expressed in multiple cancer types, accompanied by a poor prognosis. Epigenetic alterations in PLOD1 are highly heterogeneous across a wide range of tumors, and aberrant methylation and copy number variants correlate with a poor prognosis. In the tumor microenvironment, PLOD1 expression correlated positively with the infiltration level of various immunosuppressive cells (e.g., monocytes, macrophages and tumor-associated fibroblasts) and negatively with immune-killing cells (e.g., CD8^+^ T cells, B cells and CD4^+^ T cells). In addition, PLOD1 expression was associated with immune checkpoints and immunomodulatory genes. Finally, in vitro experiments demonstrated that knockdown of PLOD1 reduced the proliferation, migration and antiapoptotic abilities of T24 cells. **Conclusions:** The results of this study demonstrate that PLOD1 is a potential oncogene and prognostic biomarker in pan-cancer; tumor tissues with high PLOD1 expression reveal a relatively immunosuppressive tumor microenvironment.

## 1. Introduction

Despite the unremitting efforts of scientists and clinical doctors, cancer remains a major global public health problem [1]. According to the latest statistical assessments of epidemiology, the number of cancer deaths in the United States will reach 609,820 in 2023 [2]. In fact, the true outcome will be worse. This may be due to the coronavirus disease 2019 (COVID-19) pandemic, which hampered the diagnosis and treatment of cancer. Improving tumor immunotherapy will revolutionize the management of cancer treatment [3] but only for specific tumor types and in a limited proportion of patients, and unsatisfactory 5-year overall survival (OS) will remain [4]. Neoadjuvant immune checkpoint blockade (ICB) therapy is being widely tested in a variety of cancers, with improvements in event-free survival (EFS) and pathological complete remission (pCR) observed in the first two Food and Drug Administration (FDA)-approved studies in non-small cell lung cancer (NSCLC) and triple-negative breast cancer (TNBC) [5,6,7]. However, single-cancer-targeting studies limit our understanding of the heterogeneity of malignant tumors. Therefore, a macroscopic “pan-cancer” perspective may help us to unravel the underlying mechanisms of cancer development [8].

Cancer research has long focused on cancer cells themselves, ignoring the role of the tumor microenvironment (TME), which is an important mediator of cancer development and diagnosis; the extracellular matrix (ECM), as an important component of the TME, is more closely related to tumor progression [9,10]. Collagen, as the most abundant protein in the ECM, has a pattern of intercross-linking and precipitation that directly enhances the stiffness of tumor tissues, which, in turn, promotes tumor cell proliferation, migration, invasion and metastasis [11,12]. Procollagen-lysine, 2-oxoglutarate 5-dioxygenases (PLODs) are needed for post-translational modifications and can contribute to collagen cross-linking and extracellular matrix maturation [13]. The PLOD family consists of PLOD1, PLOD2 and PLOD3, each with different roles [14]. Among them, the PLOD1 gene is located at chromosome 1p36.2-36.3 and contains 19 exons [15]. PLOD1 hydroxylates the lysine residues in collagen, and these hydroxylysine residues form attachment points for carbohydrate units, thereby improving the stability of intermolecular cross-linking [16]. Thus, PLOD1 is also known as an enhancer of collagen fiber deposition and cross-linking stabilization. Its physiological function was discovered early in Ehlers-Danlos syndrome, a systemic disease of the conjunctive tissues with autosomal recessive inheritance [17]. In recent years, studies on PLOD1 mutations in cancer have been carried out, though not much has been reported. Nevertheless, PLOD1 has been found to be overexpressed in esophageal squamous cell carcinoma and breast cancer [18,19]. Additionally, aberrant expression of PLOD1 is associated with poorer survival in colorectal and gastric cancer patients [20]. Taken together, these findings indicate that PLOD1 may play an important role in different cancer types and subtypes.

Most previous studies have focused only on the role of PLOD1 in individual cancer types, and no pan-cancer analysis of the prognostic significance and biological function of PLOD1 has been performed thus far. Therefore, in this study, we integrated information from multiple databases and characterized the expression profiles, prognostic value, genetic mutations and functional status of PLOD1 in 33 cancer types, focusing on its potential role in tumor immunology. We also validated our bioinformatics results with in vitro experiments using bladder tumor cells. In conclusion, our study demonstrates that PLOD1 can act as an oncogene in pan-cancer, is associated with the immune response and may constitute an emerging prognostic biomarker in multiple types of cancer.

## 2. Materials and Methods

### 2.1. Gene Expression Analysis of PLOD1 at the Pan-Cancer Level

Since some tumor samples in The Cancer Genome Atlas (TCGA) database lack corresponding normal tissues, we used the UCSC XENA website (https://xenabrowser.net/datapages/, accessed on 3 February 2023) to obtain gene expression data on PLOD1 in tumors and corresponding normal samples from the TCGA database and the Genotype-Tissue Expression (GTEx) database and clinical information on a total of 33 cancers with RNA-seq data. The Cancer Cell Line Encyclopedia (CCLE) database was used to analyze the PLOD1 expression in different cancer cell lines in multidimensional studies. Information on the mutation and amplification, copy number variation (CNV) and methylation levels of PLOD1 in pan-cancer was obtained from the cBioPortal database (https://www.cbioportal.org/, accessed on 3 February 2023). Expression data were log2-transformed for further analysis. Radar plots and box plots were drawn using the R package “ggpubr” (Version number X64 3.5.1).

### 2.2. Prognostic Analysis

Survival information on survival (OS), disease-free survival (DFS), disease-specific survival (DSS) and progression-free survival (PFS) was extracted from TCGA. Patients were divided into high- and low-expression subgroups by using the median PLOD1 expression as a cutoff value in each tumor. Survival curves were plotted using the R packages “survminer” and “survival”. In addition, forest plots were used to show the results of univariate Cox regression analyses of single genes in pan-cancer, including the DFS, DSS and PFS. Forest plots were visualized using the R package “forestplot”.

### 2.3. Analysis of the Immune Microenvironment and Immune Cell Infiltration

The abundance of stromal and immune cells in the pan-cancer samples from TCGA and the association of PLOD1 with the immune cells in each cancer were calculated using the R package “ESTIMATE” and Spearman’s correlation analysis, evaluating the relationship between PLOD1 expression and the TME. Quantitative results were shown through StromalScore, ImmuneScore and TumorPurity. StromalScore, ImmuneScore, ESTIMATEScore and TumorPurity were used to show the quantitative results. Specifically, StromalScore and ImmuneScore represent the infiltration of stromal and immune cells into tumor tissues, and ESTIMATEScore is the sum of both of these, which can suggest tumor purity. We used the ImmuCellAI database (http://bioinfo.life.hust.edu.cn/ImmuCellAI, accessed on 3 February 2023) and the TIMER2 database (http://timer.comp-genomics.org/, accessed on 3 February 2023) for the correlation analysis of the immune cell infiltration. Patients with each tumor type were divided into two groups (using the median to classify PLOD1 expression into high- and low-expression groups) to compare the extent of immune cell infiltration. Finally, we analyzed the relationship between PLOD1 expression and immune-related genes, including immune checkpoints, immune-activating genes, immune-suppressing genes, chemokine receptors and chemokines.

### 2.4. Enrichment Analysis

We analyzed the proteins that bind to PLOD1 using the STRING website (https://string-db.org/, accessed on 3 February 2023), version 11.0. In addition, we screened the top 100 candidate genes with similar expression patterns to PLOD1 in pan-cancer using the TCGA website, combining PLOD1 and 20 PLOD1-binding proteins to form a gene set for GO enrichment analysis and KEGG pathway analysis.

### 2.5. Correlation Analysis Between PLOD1 Expression and the Half Inhibitory Concentration (IC50) of Anticancer Drugs

We downloaded the RNA expression profiles of tumor cell lines and IC50 information for 198 anticancer drugs from the GDSC2 database (https://www.cancerrxgene.org/, accessed on 3 February 2023). The Spearman’s correlation coefficients between PLOD1 expression and the IC50 values of anticancer drugs were calculated.

### 2.6. Cell Culture and siRNA Transfection

The T24 bladder cancer cell line was provided by the Cancer Hospital, Chinese Academy of Medical Sciences, in August 2023. The cells were authenticated in terms of the absence of mycoplasma, genotypes and morphology by Procell Life Science & Technology Co., Ltd. (Wuhan, China). The T24 cells were routinely cultured in 10% FBS (BI) DMEM (containing 1.5 mM L-glutamine, 100 U/mL penicillin and 100 μg/mL streptomycin) at 37 °C in a 5% CO_2_-saturated humidity incubator. This study was approved by the Medical Ethics Committee of the Cancer Hospital, Chinese Academy of Medical Sciences (Beijing, China).

Small interfering RNA (siRNA) was purchased from GenePharma, and the transfection process was performed according to the instructions for Lipofectamine 2000 transfection reagent (Invitrogen, Suzhou, China). The target sequences of the PLOD1 siRNA were as follows: negative control: sense (5′-3′) GCGACGAUCUGCCUAAGAUdTdT, antisense (5′-3′) AUCUUAGGCAGAUCGUCGCdTdT and antisense (5′-3′) AUCUUUAGGCAGAUCGUCGCdTdT; PLOD1-Homo965: sense (5′-3′) GCAACUACAUCCCGCGCUUTT and antisense (5′-3′) AAGCGCGGGAUGUAGUUGCTT; PLOD1-Homo1322: sense (5′-3′) GCAGCUGCACCUACUACUUTT and antisense (5′-3′) AAGUAGUAGGUGCAGCUGCTT.

### 2.7. RNA Extraction and Real-Time Quantitative PCR (RT-qPCR)

Total RNA was extracted using TRIzol reagent, and the RNA concentration was measured using a Nanodrop and then reverse-transcribed into cDNA. We performed RT-qPCR using ABI StepOne Plus. GAPDH was used as the reference gene. The following primers were used: PLOD1-F3: TGCCCGGATGTCTATTGGTT; PLOD1-R3: CTTGTTGTTGCCCAGAGACC; HGAPDH-FO: CATGAGAAGTATGACAACAGCCT; and HGAPDH-RE: AGTCCTTCCACGATACCAAAGT.

### 2.8. Western Blotting

We extracted the total protein using mixed RIPA lysis buffer. The total protein concentration was determined using the Bradford method. The samples were separated using 10% SDS-PAGE and transferred onto a membrane, which was then blocked with blocking buffer for 2 h at room temperature. The membranes were then incubated with a primary antibody (PLOD1 antibody, 1:1000) at 4 °C overnight. The next day, the membranes were co-incubated with HRP-conjugated goat anti-mouse IgG (JIR 115-035-003, 1:10,000) for 1 h at room temperature. Finally, chemiluminescence detection was performed using ECL substrate, with exposure using a Tanon 4200 (Tanon, Shanghai, China) fully automated chemiluminescence image analysis system and quantification according to grayscale analysis using Gel-Pro analyzer software (version number 4.0.00.001).

### 2.9. The CCK-8 Experiment

A CCK-8 reagent kit was purchased from DOJINDO (Tokyo, Japan). The transfected cells were inoculated into 96-well plates (Corning, Shanghai, China), mixed and incubated in an incubator, and the cell viability was measured by adding the CCK-8 reagent after 6 h. The medium was aspirated at 24 h, 48 h and 72 h, and the working solution (serum-free DMEM:CCK-8 reagent = 10:1) was added. The absorbance at 450 nm was measured using a PerkinElmer EnSpire enzyme marker.

### 2.10. The Wound-Healing Experiment

The transfected cells were inoculated into a 6-well plate (Corning). By using the tip of the pipette compared to a straightedge, a scratch on the plane of the cells that spread was formed. The cells were washed 3 times with PBS, and serum-free medium was added. The 6-well plates were placed under a microscope at 0 h and 24 h to capture images. Finally, Image-Pro Plus was used to measure the area and length of each photographed scratch, and the width of each scratch was calculated according to the value of scratch width = scratch area/scratch length value. The magnification of the microscope is 100×.

### 2.11. The Apoptosis Assay and the Cell Cycle Assay

In order to investigate the effect of PLOD1 expression on cell apoptosis, cells were collected after the corresponding treatment, digested with trypsin without EDTA, centrifuged and collected at a microcentrifuge speed of 2000 rpm for 5 min, and the culture medium was discarded. We washed the cells twice with PBS (centrifuged at 2000 rpm for 5 min) and collected 5 × 105 cells; added 400 μL of binding buffer suspension to the cells; added 5 μ L of Annexin V-FITC and 10 μL of PI staining solution (Annexin V-FITC/PI kit, Mbchem, M3021, Guangzhou, China); mixed well; and left them to react in the dark for 5–15 min at room temperature. Within 1 h, we performed flow cytometry (BD FACS Arial II) detection and analyzed the cell apoptosis rate using FlowJo software (Version number vX.0.7). Regarding the cell cycle experiment, the cells were collected by centrifugation and fixed overnight with 75% ethanol at 4 °C. After centrifugation, the cells were mixed into 100 μL of binding buffer and soaked in 10 μL of PI for 30 min in the dark. Then, we used flow cytometry to measure the samples at 488 nm.

### 2.12. Statistical Analyses

All the gene expression data were standardized by log2 transformation. Differences between two groups were assessed using unpaired *t* tests, and the data are presented as the mean ± standard deviation. The association between PLOD1 and patient prognosis was assessed using the Kaplan-Meier method, and the results are presented as risk ratios, 95% confidence intervals and P values from the log-rank test. Spearman’s correlation coefficients were used to assess the correlation between two groups. All the statistical analyses were performed using R software (version 4.1.0), except for the online website tool; *p* values < 0.05 were considered significantly different.

## 3. Results

### 3.1. Aberrant Expression of PLOD1 in Pan-Cancer

**Figure 1:**
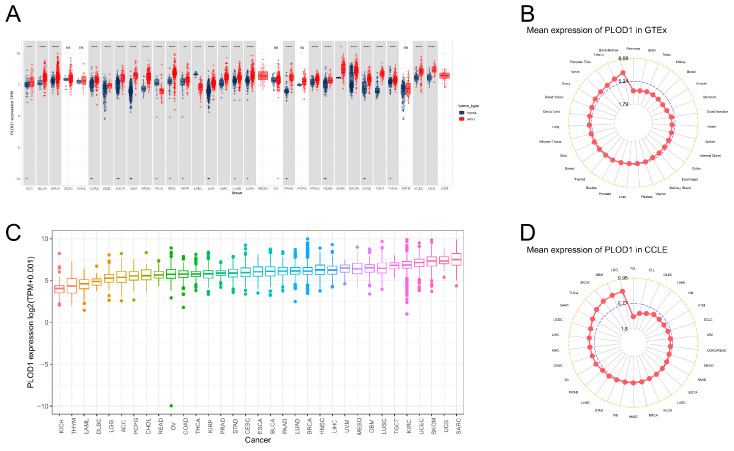
PLOD1 expression. (**A**) Pan-cancer expression profiles of PLOD1 from the TCGA and GTEx cohorts; (**B**) normal tissue expression of PLOD1 in the GTEx cohort; (**C**) tumor tissue expression of PLOD1 in the TCGA cohort; (**D**) expression of PLOD1 in the CCLE cohort in cancer cell lines. * *p* < 0.05; ** *p* < 0.01; *** *p* < 0.001; **** *p* < 0.0001, ns *p* ≥ 0.05.

To compare the PLOD1 expression between tumors and normal tissues as fully as possible, we combined data from 33 tumor types from the TCGA and GTEx databases to analyze the differences in PLOD1 expression (Figure 1A). Overall, PLOD1 was upregulated in the majority of tumor types, including adrenocortical carcinoma (ACC), bladder urothelial carcinoma (BLCA), breast cancer (BRCA), colon adenocarcinoma (COAD), diffuse large B-cell lymphoma (DLBC), esophageal carcinoma (ESCA), glioblastoma multiforme (GBM), head and neck squamous cell carcinoma (HNSC), renal clear cell carcinoma (KIRC), renal papillary cell carcinoma (KIRP), low-grade glioma (LGG), hepatocellular carcinoma (LIHC), lung adenocarcinoma (LUAD), lung squamous cell carcinoma (LUSC), pancreatic adenocarcinoma (PAAD), prostate adenocarcinoma (PRAD), rectal adenocarcinoma (READ), sarcoma (SARC), cutaneous melanoma (SKCM), gastric adenocarcinoma (STAD), testicular germ cell tumor (TGCT), thyroid carcinoma (THCA), uterine endometrial tumor (UCEC) and uterine carcinosarcoma (UCS). We also analyzed the distribution of PLOD1 in normal human tissues using the GTEx database (Figure 1B), which showed that PLOD1 was most highly expressed in the bone marrow and least expressed in the pancreas. We next assessed its expression in the TCGA pan-cancer tissues (Figure 1C), which showed that the PLOD1 expression was highest in SARC and lowest in kidney chromophobe (KICH). Finally, we found similar results in the CCLE database (Figure 1D), with PLOD1 being more highly expressed in SARC cell lines and having the highest expression in LGG cell lines.

### 3.2. Mutations in PLOD1

**Figure 2:**
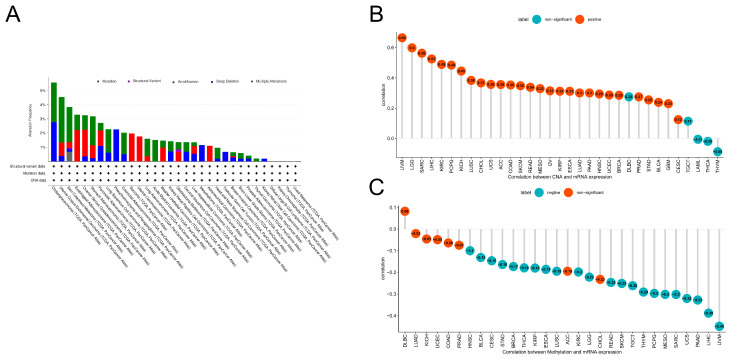
Genetic alterations in PLOD1. (**A**) cBioPortal database of mutations in PLOD1; (**B**) correlation between PLOD1 expression and copy number variation (CNV); and (**C**) correlation between PLOD1 expression and DNA methylation.

Gene mutations and epigenetic changes play a key role in regulating carcinogenesis. We therefore used the cBioPortal database to explore genetic variations in PLOD1 (Figure 2A). The results showed that PLOD1 has a mutation frequency of <5% in the vast majority of cancers, except for CHOL, in which mutation and deep deletion are the main types of mutations. Moreover, we found that the trend in PLOD1 gene mutation in SARC, uterine carcinosarcoma (UCS), SKCM, UCEC, KIRC, LUSC and GBM was consistent with its higher mRNA level. We also analyzed the association between gene expression and copy number variation (CNV) (Figure 2B). The results showed that PLOD1 mRNA expression was mainly positively correlated with CNV, with uveal melanoma (UVM) being the most significant. We then calculated the effect of DNA methylation as an epigenetic modality on PLOD1 expression (Figure 2C). There was a significant negative correlation (r ≤ −0.3) between PLOD1 expression and DNA methylation in UVM, LIHC, RAAD, UCS, SARC, mesothelioma (MESO) and pheochromocytoma and paraganglioma (PCPG). In combination with the mRNA expression analysis of PLOD1, it can be concluded that in some cancers, abnormal expression of PLOD1 may be associated with genetic alterations and altered levels of DNA methylation.

### 3.3. Prognostic Analysis

**Figure 3:**
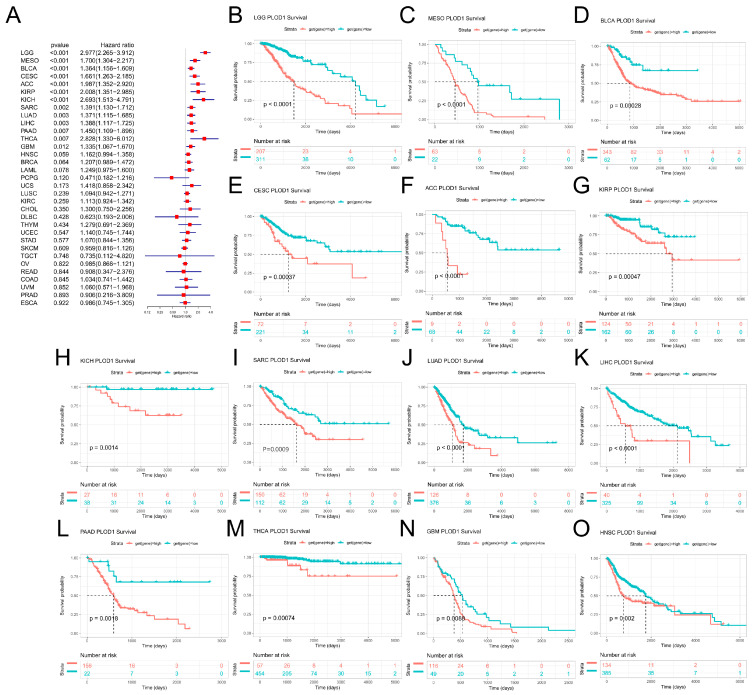
Association between PLOD1 expression and patients’ overall survival (OS). (**A**) Forest plot showing the results of univariate Cox regression analysis of PLOD1 in TCGA pan-cancer samples. (**B**–**O**) Kaplan–Meier survival curves for PLOD1 expression in tumors with significant correlation.

**Figure 4:**
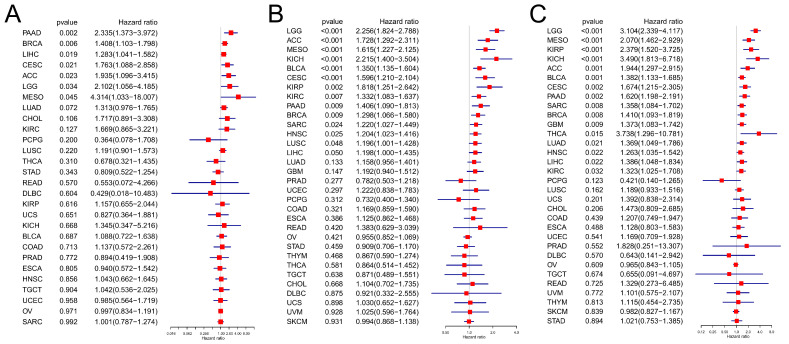
Prognostic significance of PLOD1 for patients’ disease-free survival (DFS), progression-free survival (PFS) and disease-specific survival. (**A**) DFS; (**B**) PFS; and (**C**) DSS.

We then analyzed the prognostic significance of PLOD1 in cancer patients. First, the Cox regression analysis showed that PLOD1 was a risk factor for LGG, MESO, BLCA, CESC, ACC, KIRP, KICH, SARC, LUAD, LIHC, PADD, THCA and GBM (Figure 3A). Similar results were obtained when we used a Kaplan-Meier survival analysis (Figure 3B–N), with HNSC also included (Figure 3O). PLOD1 was not found to be a protective factor for any cancer. Regarding the relationship between PLOD1 and DFS (Figure 4A), a forest plot showed that high levels of PLOD1 expression were strongly associated with poor outcomes in patients with PADD, BRCA, LIHC, CESC, ACC, LGG and MESO. The PFS analysis showed that PLOD1 can serve as prognostic risk factor for LGG, ACC, MESO, KICH, BLCA, CESC, KIRC, PADD, BRCA, SARC, HNSCH and LUSC patients (Figure 4B). Finally, to avoid bias from patients who did not die from cancer, we also analyzed the relationship between PLOD1 expression and DSS (Figure 4C). The Cox regression analysis showed that high PLOD1 expression in LGG, MESO, KIRP, KICH, ACC, BLCA, CESC, PAAD, SARC, BRCA, GBM, THCA, LUAD, HNSC, LIHC and KIRC patients was associated with a worse prognosis. The above data showed that high PLOD1 expression was significantly and positively associated with a poor prognosis in patients with various cancers.

### 3.4. Analysis of the TME and Immune Cell Infiltration

**Figure 5:**
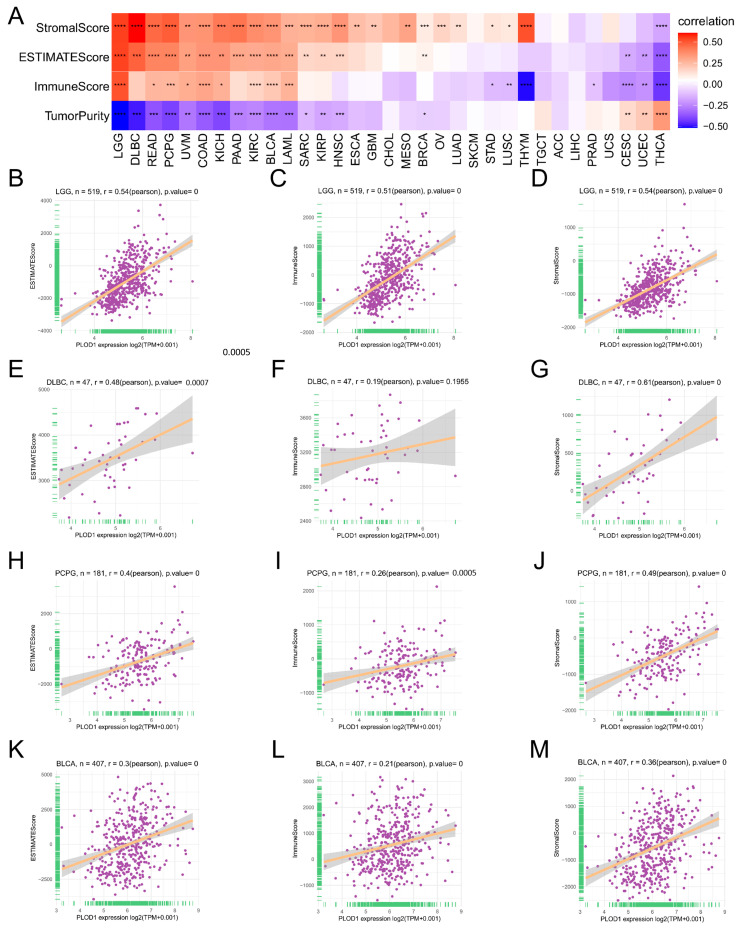
Tumor microenvironment (TME) analysis of PLOD1. (**A**) Heatmap showing the correlation between PLOD1 expression and TME score in pan-cancer. (**B**–**D**) Correlation analysis of PLOD1 expression with ESTIMATEScore, ImmuneScore and StromalScore in low-grade glioma (LGG). (**E**–**G**) Correlation analysis of PLOD1 expression with ESTIMATEScore, ImmuneScore and StromalScore in diffuse large B-cell lymphoma (DLBC). (**H**–**J**) Correlation analysis of PLOD1 expression with ESTIMATEScore, ImmuneScore and StromalScore in pheochromocytoma and paraganglioma (PCPG). (**K**–**M**) Correlation analysis of PLOD1 expression with ESTIMATEScore, ImmuneScore and StromalScore in bladder urothelial carcinoma (BLCA). * *p* < 0.05; ** *p* < 0.01; *** *p* < 0.001; **** *p* < 0.0001.

**Figure 6:**
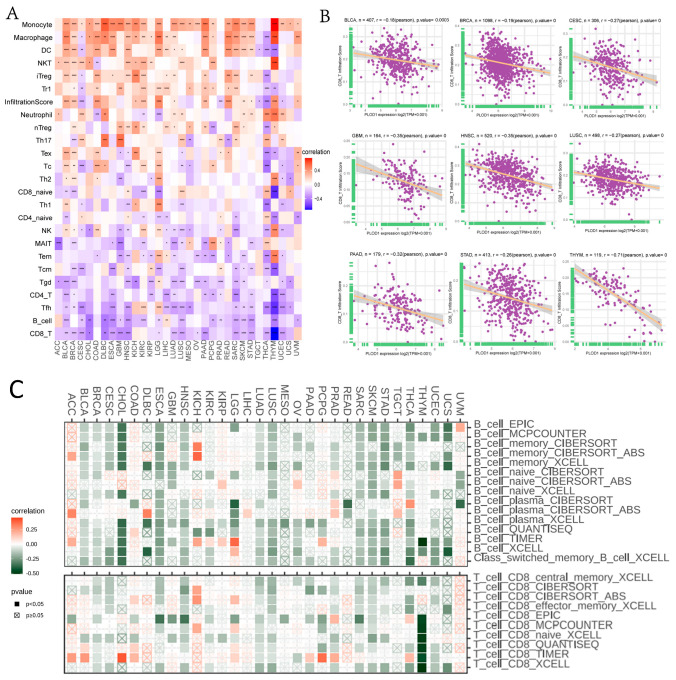
Correlation analysis between PLOD1 expression and infiltrating immune cells. (**A**) Correlation between PLOD1 expression level and the level of infiltration of 25 immune cells. (**B**) Correlation between PLOD1 expression and CD8^+^ T-cell infiltration levels using the TIMER2 database. (**C**) Correlation between PLOD1 expression and B-cell and CD8^+^ T-cell infiltration in the ImmuCellAI database. * *p* < 0.05; ** *p* < 0.01; *** *p* < 0.001; **** *p* < 0.0001.

To understand the immunomodulatory function of PLOD1, we calculated the immune and stromal scores for PLOD1 in pan-cancer using the R package “ESTIMATE” (Figure 5A). The results showed that PLOD1 was positively correlated with StromalScore, ImmuneScore and ESTIMATEScore in multiple cancer types; on the other hand, it was negatively correlated with the corresponding TumorPurity. Examples included LGG (Figure 5B–D), DLBC (Figure 5E–G), PCPG (Figure 5H–J) and BLCA (Figure 5K–M). Furthermore, we continued to analyze the relationship between PLOD1 and immune cell infiltration, which we correlated using the ImmuCellAI database and the TIMER2 database. Based on the ImmuCellAI database, PLOD1 was significantly correlated with the abundance of multiple infiltrating immune cells (Figure 6A). Among them, PLOD1 showed a positive correlation with a variety of immune cells, such as monocytes, macrophages and Treg cells. It also showed a negative correlation with a variety of immune cells, particularly CD8^+^ T cells (Figure 6B). We found similar results when using the TIME2 database (Figure 6C). PLOD1 expression was associated with a decreased abundance of CD8^+^ T cells and B cells, among others, in a variety of cancers. Taken together, the immunosuppression produced by high PLOD1 expression may partly explain its association with a poor prognosis.

**Figure 7:**
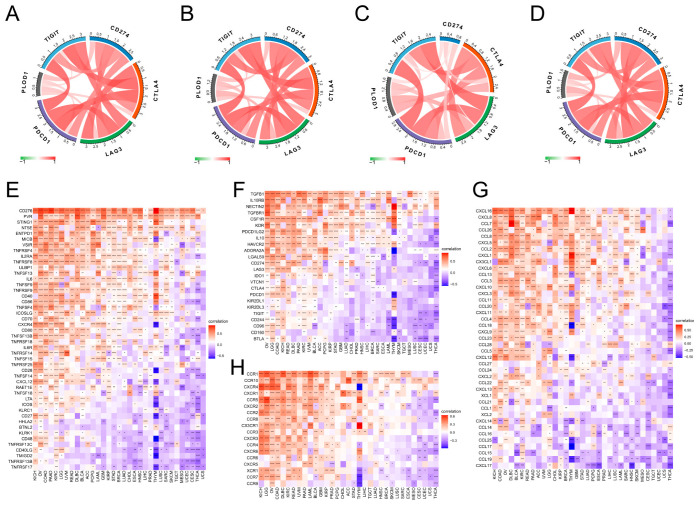
Correlation analysis between PLOD1 expression and immunomodulation-related genes. (**A**–**D**) Correlation between PLOD1 expression and immune checkpoint genes in BLCA, colon adenocarcinoma (COAD), kidney chromophobe (KICH) and rectal adenocarcinoma (READ). The red line represents positive correlation, and the green line represents negative correlation; the darker the color, the stronger the correlation. (**E**–**H**) Correlation between PLOD1 expression and immunosuppressive genes, immune-activating genes, chemokine receptors and chemokines. * *p* < 0.05; ** *p* < 0.01; *** *p* < 0.001; **** *p* < 0.0001.

Immune checkpoint inhibitor therapy targeting PD-1, PD-L1 and CTLA-4 has been approved for multiple cancer therapies. We therefore further analyzed the relationship between PLOD1 and multiple immune checkpoints, as well as immunomodulatory genes. Notably, PLOD1 was strongly positively correlated with immune checkpoint proteins such as PDCD1, CTLA4, LAG3, CD274 and TIGIT in a variety of cancers, such as BLCA, COAD, KICH and RDAD, as shown in Figure 7A–D. PLOD1 was significantly and strongly correlated with most immune-related genes in specific cancer types, as shown by the positive correlation between chemokine receptors such as CCR1, CCR10, CXCR4 and CXCR1 and chemokines such as CXCL16, CXCL8 and CCL7 and the expression of PLOD1 in a wide range of tumors (Figure 7G,H). In addition, immunosuppressive and immune-activating genes were strongly associated with PLOD1 expression in 33 cancers (Figure 7E,F).

### 3.5. Enrichment Analysis

**Figure 8:**
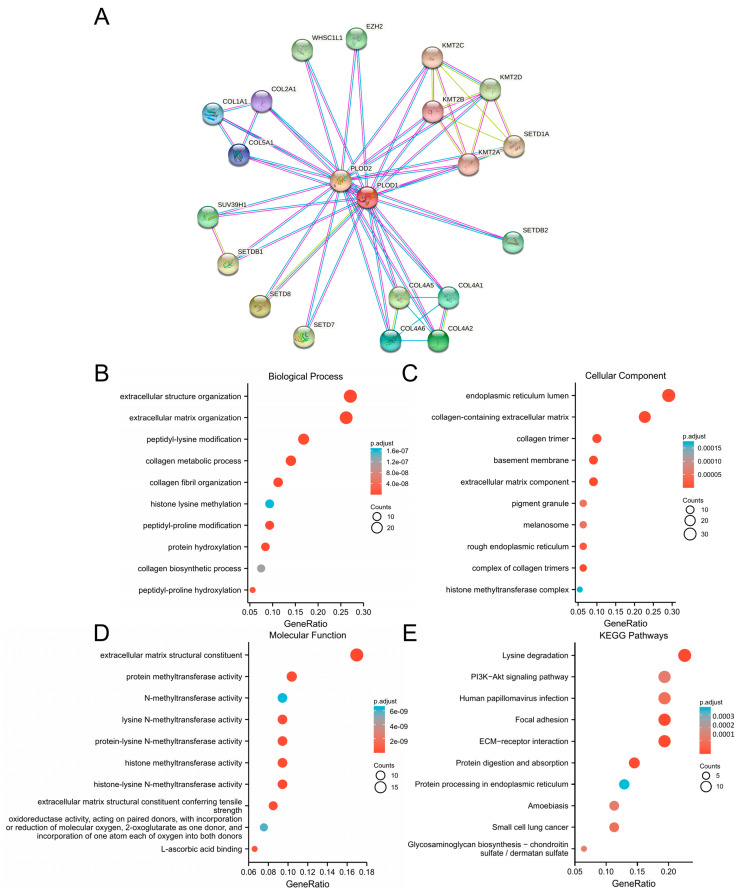
Co expression network and functional enrichment analysis of PLOD1. (**A**) PLOD1-binding proteins in the STRING database; (**B**–**E**) GO-BP, GO-CC, GO-MF and KEGG pathway analyses based on PLOD1-binding proteins and related genes.

To explore the co-expression network and enrichment pathway of PLOD1 in pan-cancer, we first predicted binding proteins for PLOD1 using the STRING website. The results showed a total of 20 proteins, including PLOD2, EZH2, WHSC1L1, COL2A1, COL1A1, COL5A1, SUV39H1, SETDB1, SETD8, SETD7, COL4A6, COL4A5, COL4A1, COL4A2, SETDB2, KMT2A, SETD1A, KMT2B, KMT2D and KMT2C (Figure 8A). Then, we conducted GO and KEGG enrichment analyses on a gene set containing 121 genes. The results indicated that these genes are involved in peptide lysine activity, processes in collagen metabolism, histone lysine metabolism, peptide proline modification, protein methyltransferase activity, N-methyltransferase activity, lysine N-methyltransferase activity, histone methyltransferase activity, oxidoreductase activity and L-ascorbic acid binding (Figure 8B–D). In addition, we found that PLOD1 is involved in tumor development through lysine degradation, the PI3K-Akt signaling pathway, focal adhesion, ECM–receiver interaction and protein digestion and absorption (Figure 8E).

### 3.6. Drug Resistance Analysis

**Figure 9:**
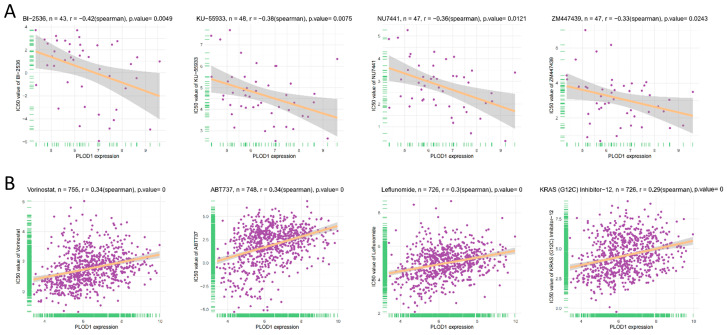
Correlation analysis of PLOD1 expression with IC50 values of anticancer drugs. (**A**) Relationship between PLOD1 expression and IC50 of significantly negatively correlated drugs; (**B**) relationship between PLOD1 expression and IC50 of significantly positively correlated drugs.

Finally, we analyzed the relationship between the half maximum inhibitory concentration (IC50) and PLOD1 for 192 anticancer drugs based on the Cancer Drug Sensitivity Genomics (GDSC2) database. The results showed that PLOD1 was negatively correlated with the IC50 values of only six drugs: BI-2536, dasatinib, RO-3306, ZM447439, NU7441 and KU-55933 (Figure 9). In contrast, the expression level of PLOD1 was positively correlated with the IC50 value of 152 drugs.

### 3.7. Functional Analysis of PLOD1 in T24 Cells

**Figure 10:**
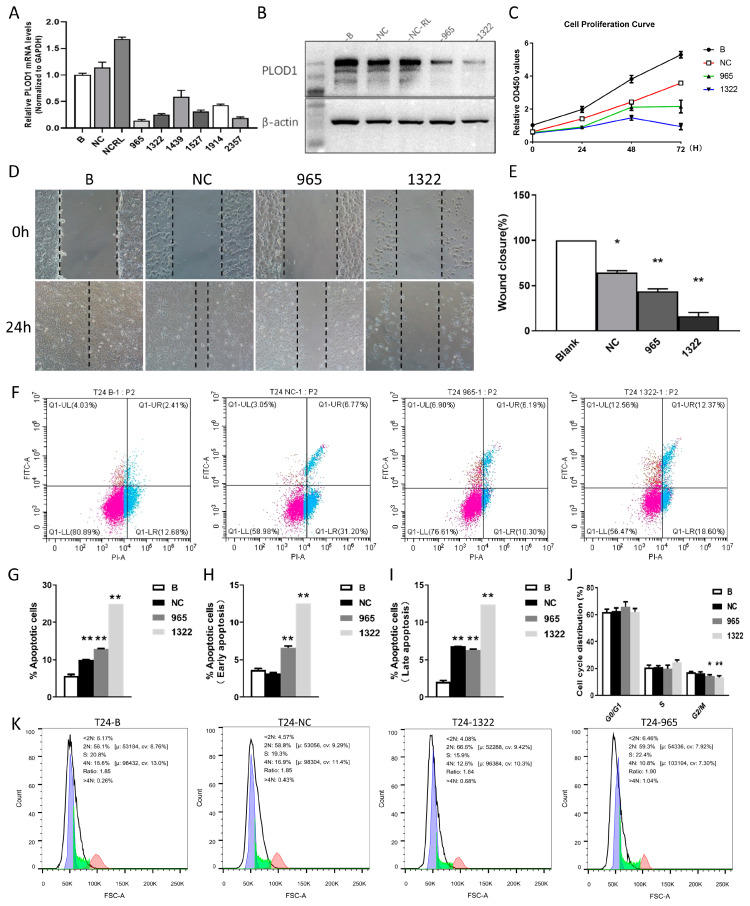
Effect of PLOD1 knockdown on function of T24 cell lines; all experiments were performed in triplicate. (**A**) RT-qPCR verification of PLOD1 knockdown efficiency in T24 cells; (**B**) Western blot verification of PLOD1 knockdown efficiency in T24 cells; (**C**) CCK-8 assay to analyze effect of PLOD1 knockdown on cellular proliferation; (**D**,**E**) cell scratch assay to analyze effect of PLOD1 knockdown on cellular healing ability; (**F**–**I**) flow cytometry to analyze changes in apoptosis; (**J**,**K**) flow cytometry to analyze changes in cell cycle progression. All the data represent the mean ± standard deviation of three independent experiments; * *p* < 0.05; ** *p* < 0.01.

The above results confirmed the powerful role of PLOD1 in a variety of cancers. Since elevated PLOD1 expression was significantly associated with a poor prognosis in BLCA, it is necessary to explore the potential role of PLOD1 in BLCA further. Table 1 shows the results of a multivariate Cox regression analysis of PLOD1 and the clinical characteristics of BLCA in TCGA. PLOD1 was associated with a higher immunotherapy response, while elevated PLOD1 also predicted a worse tumor infiltration depth (*p* = 0.023), pathological stage (*p* = 0.006) and histologic grade (*p* < 0.001). Next, we used T24 cells to analyze the biological function of PLOD1 through functional experiments. We designed six different PLOD1 siRNAs for transfecting the T24 cells, and the qPCR results showed greater knockdown efficiencies for si-965 and si-1322 (Figure 10A). The PLOD1 protein expression in the T24 cells transfected with si-965 and si-1322 was significantly reduced (Figure 10B). The CCK-8 assay was used to assess whether PLOD1 affected the proliferation ability of the T24 cells (Figure 10C). The results showed that knockdown of PLOD1 significantly inhibited the proliferation of the T24 cells. Based on the results of the scratch assay, we found that the migration ability of the transfected T24 cells was significantly decreased (Figure 10D,E). Figure 10F,G show that the total apoptosis rates of the cells with PLOD1-knockdown were 13.09% (siRNA-965) and 24.93% (siRNA-1322), respectively, significantly higher than those of the blank group (6.44%) and the control group (9.82%). The same results were obtained for early apoptosis and late apoptosis rates (Figure 10H,I). Finally, combining the cell cycle assay and the apoptosis assay, we conclude that knocking down PLOD1 has an inhibitory effect on the cell cycle (Figure 10J,K).

## 4. Discussion

Given the complexity of tumor pathogenesis, the high degree of heterogeneity and unsatisfactory therapeutic outcomes, the search for accurate and effective predictive targets to improve diagnosis and treatment has always been a common goal of clinicians and researchers, as well as the scientific community as a whole. It is known that the stability and functional expression of proteins are regulated by many processes while maintaining the normal activities of the organism [21,22]. PLOD family genes have received increasing attention due to their key role in collagen synthesis. Among them, PLOD1 is responsible for lysyl hydroxylation and acts as a specific telopeptide lysyl hydroxylase [23]. It has been demonstrated that PLOD1 catalyzes lysyl hydroxylation and directly promotes collagen cross-linking and deposition, which ultimately leads to cancer and regulates cancer cell proliferation, apoptosis, invasion and migration [24,25]. For example, in gastric cancer patients, higher PLOD1 expression is significantly associated with a shorter OS and PFS [20]. Studies have reported that PLOD1 promotes lung carcinogenesis through E2F1 activation, which also provides a novel strategy for its treatment [26]. However, the pan-cancer expression profile and diagnostic significance of PLOD1 remain incompletely characterized. Next-generation sequencing technologies and advanced data processing and analysis methods have transformed our fundamental understanding of cancer’s occurrence and progression [27]. We therefore integrated multigroup bioinformatics platforms and datasets to comprehensively analyze the pan-cancer characteristics of PLOD1. It is hoped that this study provides new insights to promote the role of PLOD1 in the diagnosis and treatment of cancer.

Previous studies have shown that dysregulation of PLOD1 is closely associated with tumorigenesis and malignant progression. For example, the PLOD1 mRNA and protein expression in clear cell renal carcinoma (ccRCC) tissues is significantly higher than that in normal tissues, which is also accompanied by a high pathological grade, advanced tumor staging and a poor OS [28]. Elevated PLOD1 in osteosarcoma tissues is associated with distant metastasis, Enneking stage and a worse prognosis. Simultaneous silencing of PLOD1 leads to inhibition of the propagation, motility and invasive ability of MG63 cells and U-20S cells [26,29]. Based on the above findings, we comprehensively explored the PLOD1 expression in different cancers. Differential expression results showed statistically significant upregulation of PLOD1 expression in a total of 24 of 33 cancers compared to adjacent healthy tissues. Moreover, high expression of PLOD1 was accompanied by poor prognostic outcomes. Our results revealed that high PLOD1 expression predicted poor overall survival in a variety of tumors, especially LGG, MESO, BLCA, CESC and ACC. Similarly, high PLOD1 expression remained an unfavorable indicator for tumor patients in the DSS, DFS and PFS analyses. This is consistent with the previously reported role of PLOD1 in colorectal cancer, osteosarcoma and tongue squamous cell carcinoma [29,30,31]. These findings suggest that PLOD1 is an independent molecular marker associated with clinical features.

Factors affecting gene expression are classified into epigenetic and genetic factors [32]. However, studies on PLOD1 tumor-associated mutations are still relatively scarce. In our study, we observed that for most tumors, PLOD1 exhibited a maximum mutation rate of approximately 5%, with point mutation as its major mode of mutation. At the epigenetic level, the promoter methylation levels of PLOD1 were generally downregulated in a wide range of cancers. Aberrant DNA methylation patterns in cancer cells are widely recognized phenomena that can often repress gene expression by altering the chromatin structure, DNA conformation and DNA stability and play an important role in tumorigenesis [33,34]. This study found a significant negative correlation between the methylation level of PLOD1 and mRNA expression, which suggests that its methylation may be one of the reasons for the aberrant expression of PLOD1. In addition, CNV is an important component of genomic structural variation, which can affect the expression of coding and noncoding genes, as well as the activity of various signaling pathways [35]. The CNV analysis revealed that the frequency of PLOD1 gene copy number variation was highly heterogeneous. These results suggest that blocking the aberrant expression of PLOD1 at the genetic and epigenetic levels may be a therapeutic modality to reverse tumorigenesis.

Growing evidence suggests that the tumor microenvironment (TME) is significantly associated with tumor prognosis [36]. The TME consists of the extracellular matrix, the vascular network, lymphatic vessels, cells and soluble molecules. Most of the tumor tissue is encapsulated by infiltrating stromal cells and immune cells [37]. Research has found that immune scores can serve as indicators of survival, recurrence and drug resistance in cancer patients [38]. Our study confirms that PLOD1 was positively correlated with immune scores and stromal scores in most tumors, including LGG, DLBC, READ and BLCA. This suggests that PLOD1 interacts with tumor cells and tumor-infiltrating immune cells. Tumor-infiltrating immune cells have been shown to be closely associated with tumor growth, invasion and metastasis [39]. Here, we report a statistical association between PLOD1 expression and infiltrating immune cells for the first time. We found that in 33 cancers, PLOD1 expression was negatively correlated with CD8^+^ T cells, B cells and CD4^+^ T cells, among others, and positively correlated with monocytes, macrophages, neutrophils and tumor-associated fibroblasts (CAFs). Previous studies have shown that CAFs are the most abundant tumor stromal cells in the TME, and these cells secrete various cytokines and metabolites and shape the barrier through the extracellular matrix, ultimately inhibiting the function of immune cells and drug uptake to induce cancer cell proliferation [40]. Neutrophil adhesion to fibronectin may lead to the release of hydroxylysine into the extracellular space, which further affects cell morphology and the cytoskeleton [41]. Additionally, neutrophils enable tumor metastasis and cell cycle progression by escorting circulating tumor cells [42]. In contrast, CD8^+^ T cells function as killer cells, dominate antitumor immunity and greatly influence the outcomes of cancer immunotherapy [43]. This may also explain the association of high PLOD1 expression with a poor prognosis in most tumors. Due to their unique role in immune escape, immune checkpoints have become a major target for immunotherapy-related drug development [44]. Our study found that PLOD1 was positively correlated with the expression of several immune checkpoint genes, which may represent a better response to immunotherapy. However, PLOD1 is also associated with a large number of immune modulator genes, especially certain chemokines and chemokine receptors. These chemokines recruit immunosuppressive cells into the TME, creating an immunosuppressive environment that can undermine immunotherapy. The regulatory network between PLOD1 and the components of the immune microenvironment is complex and varied; therefore, more clinical trials are needed to explore the role PLOD1 plays in immunotherapy.

There is no doubt that PLOD1 plays an important role in cancer development, but the underlying mechanisms remain unclear. We found that PLOD1 is mainly involved in the composition of the extracellular matrix through an enrichment analysis of genes co-expressed with PLOD1. As previously reported, PLOD1 regulates the hydroxylation of lysyl residues in type V collagen, a protein that acts as an ECM component in the connective tissues to support cellular integrity and regulate cell signaling [45]. We hypothesized that PLOD1 might be involved in the remodeling or degradation of the extracellular matrix and might also play a role in tumor invasion. We eventually verified this hypothesis using in vitro functional experiments.

Our study still has some limitations that need to be addressed. First, our pan-cancer study on PLOD1 was limited to bioinformatics analyses and in vitro experiments and lacked large-scale patient cohort studies. Second, given that our data analysis relied on multiple online databases, there may have been systematic bias, which may have affected the accuracy of the final results. Third, this study of PLOD1 is not deep enough and lacks specific mechanistic pathway studies. We will continue to conduct follow-up studies.

## 5. Conclusions

In summary, our study demonstrated that PLOD1 is commonly highly expressed in a wide range of cancers, which is accompanied by poor clinical prognostic outcomes, with an emphasis on the fact that we confirmed the complex relationship between PLOD1 and the tumor immune microenvironment. We have provided clues for further research on the specific mechanisms of PLOD1 in cancer progression and treatment.

## Figures and Tables

**Table 1:** Multivariate Cox regression analysis of clinical characteristics of PLOD1 and BLCA in TCGA.

Characteristics	Low Expression of PLOD1	High Expression of PLOD1	*p* Value
*n*	206	206	
Pathologic T stage, n (%)			0.023
T1	4 (1.1%)	1 (0.3%)	
T2	68 (18%)	50 (13.2%)	
T3	83 (22%)	113 (29.9%)	
T4	32 (8.5%)	27 (7.1%)	
Pathologic stage, n (%)			0.006
Stage I	4 (1%)	0 (0%)	
Stage II	77 (18.8%)	52 (12.7%)	
Stage III	64 (15.6%)	78 (19%)	
Stage IV	59 (14.4%)	76 (18.5%)	
Gender, n (%)			0.502
Female	51 (12.4%)	57 (13.8%)	
Male	155 (37.6%)	149 (36.2%)	
Primary therapy outcome, n (%)			0.024
PD	25 (7%)	45 (12.7%)	
SD	14 (3.9%)	16 (4.5%)	
CR	131 (36.9%)	102 (28.7%)	
PR	12 (3.4%)	10 (2.8%)	
Age, n (%)			0.551
≤70	119 (28.9%)	113 (27.4%)	
>70	87 (21.1%)	93 (22.6%)	
Histologic grade, n (%)			<0.001
High-grade	183 (44.7%)	205 (50.1%)	
Low-grade	20 (4.9%)	1 (0.2%)	
Smoker, n (%)			0.279
No	60 (15%)	49 (12.3%)	
Yes	142 (35.6%)	148 (37.1%)	
OS event, n (%)			<0.001
Alive	132 (32%)	98 (23.8%)	
Dead	74 (18%)	108 (26.2%)	

## Data Availability

The analyzed datasets generated during the present study are available.
